# Risk of repeated self-harm and associated factors in children, adolescents and young adults

**DOI:** 10.1186/s12888-016-1120-2

**Published:** 2016-11-24

**Authors:** Marco Bennardi, Elaine McMahon, Paul Corcoran, Eve Griffin, Ella Arensman

**Affiliations:** 1 National Suicide Research Foundation, University College Cork, Cork, Ireland; 2 Department of Epidemiology and Public Health, University College Cork, Cork, Ireland

**Keywords:** Self-harm, Repeated self-harm, Young people, Emergency departments, Self-harm methods

## Abstract

**Background:**

Repeated self-harm represents the single strongest risk factor for suicide. To date no study with full national coverage has examined the pattern of hospital repeated presentations due to self-harm among young people.

**Methods:**

Data on consecutive self-harm presentations were obtained from the *National Self-Harm Registry Ireland.* Socio-demographic and behavioural characteristics of individuals aged 10–29 years who presented with self-harm to emergency departments in Ireland (2007–2014) were analysed. Risk of long-term repetition was assessed using survival analysis and time differences between the order of presentations using generalised estimating equation analysis.

**Results:**

The total sample comprised 28,700 individuals involving 42,642 presentations. Intentional drug overdose was the most prevalent method (57.9%). Repetition of self-harm occurred in 19.2% of individuals during the first year following a first presentation, of whom the majority (62.7%) engaged in one repeated act. Overall, the risk of repeated self-harm was similar between males and females. However, in the 20–24-year-old age group males were at higher risk than females. Those who used self-cutting were at higher risk for repetition than those who used intentional drug overdose, particularly among females. Age was associated with repetition only among females, in particular adolescents (15–19 years old) were at higher risk than young emerging adults (20–24 years old). Repeated self-harm risk increased significantly with the number of previous self-harm episodes.

Time differences between first self-harm presentations were detected. Time between second and third presentation increased compared to time between first and second presentation among low frequency repeaters (patients with 3 presentations only within 1 year following a first presentation). The same time period decreased among high frequency repeaters (patients with at least 4 to more than 30 presentations).

**Conclusion:**

Young people with the highest risk for repeated self-harm were 15–19-year-old females and 20–24-year-old males. Self-cutting was the method associated with the highest risk of self-harm repetition. Time between first self-harm presentations represents an indicator of subsequent repetition. To prevent risk of repeated self-harm in young people, all individuals presenting at emergency departments due to self-harm should be provided with a risk assessment including psychosocial characteristics, history of self-harm and time between first presentations.

## Background

Self-harm is relatively common among young people with prevalence rates in adolescent samples which range from 6.9 to 15.9% [1–4]. One review of the literature showed that 13.2% of adolescents reported engaging in self-harm at some stage in their lives, with 11.2% who reported engaging in self-harm in the last 6 months [5].

Self-harm is ‘an act with non-fatal outcome in which an individual deliberately initiates a non-habitual behaviour, that without intervention from others will cause self-harm, or deliberately ingests a substance in excess of the prescribed or generally recognised therapeutic dosage, and which is aimed at realising changes that the person desires via the actual or expected physical consequences’ [6]. This definition was developed by WHO/Euro Multicentre Study Working Group and associated with the term ‘parasuicide’ (superseded by the term ‘deliberate self-harm’, and this latter by ‘self-harm’), and includes acts involving varying levels of suicidal intent by adopting the principle that suicidal intent is on a continuum and not an all-or-nothing phenomenon [7, 8].

Research has shown that risk of suicide is associated with self-harm [9], in particular with repeated self-harm [10, 11]. A previous study showed that among 10–24-year-old males, the risk of suicide within 1 year following a single self-harm episode was 2.1%, whereas among those with repeated self-harm the risk was 4.1% [11]. Among females, a greater difference was identified, the risk of suicide for those with a single self-harm episode was 0.3% whereas among those with repeated self-harm it was 1.9% [11]. History of self-harm is not associated only with suicide but also with future repeated self-harm [12]. In addition to history of self-harm, other factors are also associated with increased risk of repetition in young people including alcohol and drug misuse, depression [12], psychiatric treatment [13], behavioural problems, disturbed family relationships, alcohol dependence in the family, social isolation, and a poor school record [12]. Repetition among young people is also associated with older age and self-cutting as method of self-harm [13].

Research investigating hospital presentations due to self-harm revealed that self-harm repetition occurred in 27.3% of people aged 10–18 years who had previously presented to emergency departments with self-harm [13]. Moreover, a repeat self-harm episode is most likely to occur in the first months following a hospital presentation [14–16].

There have been few studies on self-harm repetition in young people including a large sample and more than one centre [13]. To our knowledge, there are no studies focusing on young people with full national coverage. No studies have analysed time between first hospital presentations in young people and its association with subsequent self-harm.

This study has been conducted to investigate the pattern of self-harm presentations and repetition, in order to better understand self-harm in young people. The overall objective was to identify risk factors for long-term repetition of self-harm among children, adolescents and young adults who have presented to hospital emergency departments in Ireland with self-harm. Specifically, we aimed to identify age group similarities and differences in risk of self-harm repetition, with the focus on frequency of repetition and time between self-harm presentations. We also aimed to examine potential risk factors for repeated self-harm, such as gender, age, frequency of repetition, involvement in substance use, previous self-harm and recommended next care. We hypothesised these factors to be significantly associated with self-harm repetition according to the findings of a previous study analysing all age groups [17]. Additionally, we conducted a number of exploratory analysis with the aim to identify possible age-group and gender differences in the association between self-harm repetition and individual variables (e.g. age-group differences in the association between recommended next care following a self-harm presentation and self-harm repetition).

We conducted this study with the aim of gaining a greater understanding of risk of repeated self-harm, which is needed to inform health care services dealing with youth self-harm at hospital emergency departments.

## Methods

### Setting and sample

Data for this study were drawn from the National Self-Harm Registry Ireland. Data were extracted for consecutive patients aged 10 to 29 years who attended any of the emergency departments (ED) in the Republic of Ireland (estimated population: 4,593,300 in 2013) in consequence of non-fatal self-harm between 1st January 2007 and 31st December 2014. In total, self-harm data were collected for all 35 acute hospitals in Ireland. A small number of hospital emergency departments were re-designed (Model 2 status hospitals, HSE’s Securing the Future of Smaller Hospitals framework) [18]. The analyses included data from 1st January 2007 as full coverage of EDs of Ireland was obtained from that time onwards. A new and more precise procedure to identify self-harm cases was also adopted in 2007. The end of the study period was 31^st^ December 2014.

The definition of self-harm from the WHO/Euro Multicentre Study Working Group was used in this study [6]. All methods of self-harm are included such as intentional drug overdoses, alcohol overdoses or lacerations. Episodes involving acts without the intention to self-harm were excluded (e.g. one individual who is ill and takes additional medication without any intention to deliberately harm her/himself).

The four age group categories used were defined as follows: Children (CH) were referred to as those between 10 and 14 years, adolescents (AD) to those between 15 and 19 years, younger emerging adults (YEA) to those between 20 and 24 years, and older emerging adults (OEA) to those between 25 and 29 years.

For the purpose of statistical analysis, those individuals who had more than one presentation were categorised in two groups. *Low frequency repeaters* were referred to as those with three presentations, and *high frequency repeaters* to those with at least four presentations, within 12 months following a first presentation. A similar categorisation (frequent and infrequent repeaters) was used by C Haw, et al. [19].

Method of self-harm was recorded according the Tenth Revision of the WHO’s International Classification of Diseases codes for intentional injury [20]. The main methods were grouped as follows: overdose of drugs and medications (X60-X64), self-poisonings by alcohol (X65), poisonings which involve the ingestion of chemicals, noxious substances, gases and vapours (X66-X69), hanging (X70), drowning (X71), and self-harm by sharp object (X78). Some individuals have used more than one method at one time. In this study, methods were categorised as follows: drug overdose (only), self-cutting (only), drug overdose and self-cutting, attempted hanging, attempted drowning, other methods (which includes either methods other than those listed above or a non-common combination of methods).

Self-harm repetition was defined as a presentation at a hospital emergency department due to a self-harm act following a previous self-harm presentation.

A previous study showed that the patterns of self-harm repetition of males and females presenting at EDs in Ireland differed [17]. Therefore, in the survival analyses examining the risk of repetition, female and male patients’ presentations were analysed separately.

### Data collection

Data were entered into a computerised system by the Registry’s data registration officers. In each one of the four HSE regions (Dublin/Midlands, Dublin/North East, South, West region) a team of people collect the data that form the national dataset. A self-harm act was recorded dependent on the standardised application of the case-definition. High levels of agreement were found between the data registration officers in term of case ascertainment [17].

Encrypted patient initials, date of birth and gender were the data items used to distinguish between patients and to identify repeat presentations by the same patients.

### Statistical analyses

The following variables were included in the analyses: age, gender, method of self-harm, date of presentation, alcohol use (detected when presenting in ED), recommended next care following treatment in the hospital ED.

Univariate analyses examining associations between the total number of presentations by person and socio-demographic and method characteristics were conducted using chi-squared tests.

Associations between repetition of self-harm and socio-demographic, history of previous self-harm, method characteristics and recommended next care were examined using survival analysis. In the first step, a model was built for each of the variables. In the second step, all the variables with a *p*-value <0.10 (value present either in the male or female model) were included in a subsequent multivariate model. The survival analysis method used was the Cox proportional hazard model, in particular the conditional risk set model. The assumption of this model, proposed by Prentice, Williams and Peterson, is that an individual is not at risk of a second event until the first event has occurred and so forth. Additionally, according to these methods after the occurrence of a first failure, an individual still remains at risk of a second and subsequent failures [21–23]. Time to the next self-harm presentation was measured for the second or subsequent presentation. The analysis was stratified by failure order. Socio-demographic and self-harm method characteristics examined were age, gender, alcohol involvement, and, where applicable previous self-harm. A variable “status” was generated and included observations to next presentation, ending in an event (coded as status = 1) and observations to the end of 1-year follow-up, ending as censored (status = 0). Censored cases were all non-repeaters’ presentations and the last presentation of repeaters (to the end of the follow up). To be certain of accounting for the first presentation, people whose first presentation occurred in 2010 and onwards only were included. Follow-up was 1 year following the individual’s index presentation made between 2010 and 2013. A similar method was applied by Perry et al. [17].

Finally, we included in the survival analyses the first six presentations by subject. This cut-off was used as most of individuals who repeated had no more than six presentations, and this approach is consistent with previous studies [17, 24, 25]. The order of presentation as stratification was not included in the analysis, as it would not have allowed the performance of the whole analysis. In order to conduct the analysis including all the variables, we decided to exclude it, as this did not change the overall picture and in general the results.

Lastly, performing a generalised estimating equation model, time differences between first order presentations were analysed, adopting the same inclusion criteria used for survival analyses. For this analysis, those who repeated self-harm were categorised into two groups, based on the frequency of their repetition as *low frequency repeaters*, and *high frequency repeaters* (the definition of these categories was outlined in section Setting and sample). In particular, we compared the time between a 1st and 2nd presentation, to that between 2nd and 3rd presentation.

For the analysis STATA version 12.0 for Windows [26] and IBM SPSS version 22.0 for Windows [27] were used.

## Results

The total sample comprised 28,700 individuals aged 10–29 years. During the study period, these 28,700 individuals were involved in 42,642 self-harm episodes resulting in presentation to any of the 35 emergency departments (EDs) in Ireland. Overall, there were more females (*N* = 15,560, 54.2%) than males (*N* = 13,140, 45.8%; *χ* ^2^ = 27.6, *p* = <0.001; Table 1). At first presentation, there were 1726 (6.0%) children (CH), 9911 (34.5%) adolescents (AD), 9510 (33.1%) younger emerging adults (YEA) and 7553 (26.3%) older emerging adults (OEA). Comparing all of the age groups according to the number of presentations, a similar proportion of those who presented 1–3 times were AD (33.7%) and YEA (33.3%). The highest proportion of those who presented 4 or more times were YEA (38.4%; *χ* ^2^ = 559.99, *p* = <0.001). The majority of these episodes involved drug overdose, with self-cutting the second most common method (Table 2).

**Table 1:** Total number of self-harm presentations by gender

	One	Two	Three	Four	Five	Six	Seven/More	Total	*χ* ^2^
Male (*N*)	10,271	1621	588	259	120	82	199	13,140	
%	45.2	46.9	51.8	50.7	46.2	45.6	47.7	45.8	
Female (*N*)	12,473	1832	547	252	140	98	218	15,560	
%	54.8	53.1	48.2	49.3	53.9	54.4	52.3	54.2	
Total (*N*)	22,744	3453	1135	511	260	180	417	28,700	27.6, *p* = <0.001

**Table 2:** Self-harm presentations by method

	One	Two	Three	Four	Five	Six	Seven/More	Total
Drug overdose only (*N*)	14,296	4053	1815	1068	628	527	2297	24,684
%	62.9	58.7	53.3	52.3	48.3	48.8	44.5	57.9
Self-cutting only (*N*)	4070	1391	756	491	315	295	1607	8925
%	17.9	20.1	22.2	24.0	24.2	27.3	31.1	20.9
Overdose & self-cutting (*N*)	1033	421	220	149	103	75	414	2415
%	4.5	6.1	6.5	7.3	7.9	6.9	8	5.7
Attempted hanging only (*N*)	993	307	170	88	66	49	189	1862
%	4.4	4.5	5.0	4.3	5.0	4.5	3.7	4.4
Attempted drowning only (*N*)	371	122	81	52	20	26	87	759
%	1.6	1.8	2.4	2.5	1.5	2.4	1.7	1.8
Other (*N*)	1981	612	363	196	168	108	569	3997
%	8.7	8.9	10.7	9.6	12.9	10.0	11.0	9.4
Total (*N*)	22,744	6906	3405	2044	1300	1080	5163	42,642

### Self-harm repetition

Those individuals whose first presentation occurred during 2010–2013 (*N* = 13,736; 46.3% males and 53.7% females) were followed up for 1 year until 2013. Repetition occurred in 19.2% (*N* = 2630) of these individuals. The majority of individuals who repeated had one repetition only (62.7%, *N* = 1650). A number of those who repeated had two (19.3%, *N* = 508) or three repetitions (7.3%, *N* = 193). Moreover, 10.6% of all individuals who repeated had at least four repetitions. Finally, 4.5% (*N* = 118) had more than 6 repetitions with a wide variation in the number of episodes.

The proportion of females and males who repeated at least once was 52.6 and 47.4% respectively. By age group, the proportion of individuals repeating among CH, AD, YEA, and OEA was 24.1, 20.9, 18.6 and 16.3% respectively.

#### Cox hazard survival analysis: univariate analyses

Age, gender, method used, previous self-harm episodes, and recommended next care were examined separately using cox proportional hazard survival analysis (Table 3).

**Table 3:** Cox proportional hazard survival analysis for time to self-harm repetition from first presentation occurred during 2010–2013 - Univariate analysis

Univariate				
	Males		Females	
Variables	Hazard Ratio (95% CI)	*p*	Hazard Ratio (95% CI)	*p*
Age group				
Adolescents	1		1	
Children	0.73 (0.51–1.08)	ns	1.12 (0.91–1.37)	ns
Younger emerging adults	0.98 (0.84–1.15)	ns	0.86 (0.73–1.01)	0.06
Older emerging adults	0.93 (0.79–1.11)	ns	0.99 (0.82–1.19)	ns
Method				
Drug overdose only	1		1	
SC only	1.26 (1.09–1.45)	0.002	1.95 (1.70–2.23)	<0.001
Overdose & SC	1.47 (1.18–1.83)	0.001	1.71 (1.39–2.07)	<0.001
Attempted hanging only	0.83 (0.66–1.06)	ns	1.37 (1.02–1.84)	0.003
Attempted drowning only	0.87 (0.61–1.26)	ns	1.61 (1.10–2.34)	0.001
Other	1.19 (1.02–1.41)	0.03	1.61 (1.35–1.93)	<0.001
Previous SH presentation				
None	1		1	
One	2.69 (2.38–3.05)	<0.001	2.45 (2.16–2.77)	<0.001
Two	3.67 (3.09–4.35)	<0.001	4.94 (4.23–5.78)	<0.001
Three	5.53 (4.43–6.89)	<0.001	7.41 (6.12–8.96)	<0.001
Four	6.5 (4.91–8.59)	<0.001	8.44 (6.67–10.68)	<0.001
Five	7.9 (5.58–11.31)	<0.001	9.7 (7.27–12.93)	<0.001
Recommended next care				
General admission	1		1	
Psychiatric admission	0.99 (0.65–1.53)	ns	1.94 (1.25–3.01)	0.003
Refused to be admitted	0.83 (0.32–2.11)	ns	0.57 (0.14–2.29)	ns
Left before admission	1.05 (0.72–1.54)	ns	1.73 (1.18–2.54)	0.005
Not admitted	0.90 (0.68–1.21)	ns	1.34 (1.04–1.73)	0.03
Left without being seen	1.58 (0.98–2.55)	0.06	1.28 (0.67–2.42)	ns
Alcohol use				
Yes	1		1	
No	0.94 (0.84–1.04)	ns	0.85 (0.75–0.96)	0.007

#### Self-harm method

Among males, those using drug overdose (only) were at lower risk of repetition compared to those using self-cutting (only), overdose and self-cutting, and other methods (a rare method or combination of methods). Among females, those who used drug overdose (only) were at lower risk than those who used self-cutting (alone or combined with drug overdose), attempted hanging, attempted drowning, and other methods.

The risk of repeated self-harm among those who used self-cutting, self-cutting and overdose, attempting hanging or drowning, and other methods, compared to drug overdose (only) was higher among females, but not among males.

#### History of self-harm

History of self-harm represents a risk factor for repeated self-harm, with the risk increasing with the number of previous episodes. This association was stronger among females compared to males (Table 3).

#### Recommended next care

Comparing males of all age groups on next care following a self-harm episode revealed that the risk of repeated self-harm was similar regardless of what the recommended next care was (Table 3). However, according to some exploratory analyses, among young male emerging adults, those who had left without being seen were at higher risk of repetition than those who were admitted to general ward (HR = 0.36; CI (95%) = 0.16–0.81; *p* = 0.01) and those who were not admitted (HR = 0.42; CI (95%) = 0.22–0.81; *p* = 0.01).

Comparing females on recommended next care revealed that those who were admitted to general ward were at lower risk of repeated self-harm within 1 year (following the index presentation) compared to those who were admitted to psychiatric ward, had left before admission, and were not admitted (Table 3). This pattern was particularly strong among older female emerging adults; those who were admitted to general ward were at lower risk of repetition within 1 year (following the index presentation) compared to those who were admitted to psychiatric ward (HR = 3.31; CI (95%) = 1.20–9.16; *p* = 0.02), had left before admission (HR = 3.58; CI (95%) = 1.48–8.64; *p* = 0.005), and were not admitted (HR = 2.11; CI (95%) = 1.05–4.26; *p* = <0.04).

#### Alcohol involvement

Overall, among females the risk for self-harm repetition was lower among those who had taken alcohol compared to those who had not (Table 3). This pattern was observed only among young female emerging adults (HR = 0.81; CI (95%) = 0.66–0.99; *p* = 0.04).

#### Cox hazard survival analysis: multivariate analysis

The multivariate analysis (Table 4) identified age as a risk factor for repetition only among females, in particular AD were at higher risk than YEA. Additionally, those individuals using self-cutting (alone or combined with drug overdose) were at higher risk than those using drug overdose (alone) among females. Self-harm methods and repetition were marginally associated among males (Table 4). Previous self-harm was a risk factor for repeated self-harm, particularly among women. Risk of repetition was higher among those females who did not use alcohol.

**Table 4:** Cox proportional hazard survival analysis for time to self-harm repetition from first presentation occurred during 2010–2013 – Multivariate Analysis

Multivariate				
	Males		Females	
Variables	Hazard Ratio (95% CI)	*p*	Hazard Ratio (95% CI)	*p*
Age group				
Adolescents	1		1	
Children	1.28 (0.73–2.21)	ns	1.24 (0.92–1.67)	ns
Younger emerging adults	0.97 (0.75–1.24)	ns	0.73 (0.58–0.93)	0.01
Older emerging adults	1.03 (0.81–1.32)	ns	0.89 (0.67–1.18)	ns
Method				
Drug overdose only	1		1	
Self-cutting only	1.29 (0.98–1.69)	0.07	1.59 (1.23–2.05)	<0.001
Overdose & self-cutting	1.04 (0.67–1.61)	ns	2.09 (1.49–2.93)	<0.001
Attempted hanging only	0.94 (0.63–1.41)	ns	1.14 (0.61–2.15)	ns
Attempted drowning only	1.31 (0.66–2.59)	ns	0.89 (0.38–2.11)	ns
Other	1.16 (0.88–1.54)	ns	1.47 (1.11–1.97)	0.009
Previous SH presentation				
None	1		1	
One	2.67 (2.11–3.38)	<0.001	2.11 (1.65–2.7)	<0.001
Two	3.13 (2.27–4.31)	<0.001	5.23 (3.91–6.99)	<0.001
Three	5.21 (3.65–7.42)	<0.001	6.63 (4.81–9.17)	<0.001
Four	4.97 (3.01–8.21)	<0.001	6.95 (4.53–10.67)	<0.001
Five	4.73 (2.47–9.06)	<0.001	9.43 (5.59–15.88)	<0.001
Recommended next care				
General admission	1		1	
Psychiatric admission	0.91 (0.61–1.39)	ns	1.21 (0.78–1.88)	ns
Refused to be admitted	1.07 (0.49–2.31)	ns	0.59 (0.15–2.23)	ns
Left before admission	1.00 (0.68–1.46)	ns	1.48 (0.98–2.23)	0.06
Not admitted	0.89 (0.67–1.18)	ns	1.20 (0.93–1.54)	ns
Left without being seen	1.47 (0.89–2.42)	ns	1.21 (0.66–2.24)	ns
Alcohol use				
Yes	1		1	
No	0.81 (0.64–1.01)	0.06	0.78 (0.61–1.01)	0.05

Overall, risk of repetition was similar regardless of the recommended next care. Additional exploratory multivariate analyses revealed that young male emerging adults who were admitted to general ward were at lower risk for repetition compared to those who had left without being seen (HR: 2.32; CI (95%) = 1.03–5.24; *p* = 0.004). Moreover, older female emerging adults admitted to general ward were at lower risk for repetition compared to those who refused to be admitted (HR = 2.89; CI (95%) = 1.00–8.32; *p* = <0.001), had left before admission (HR = 4.83; CI (95%) = 2.07–11.27; *p* = <0.001), were not admitted (HR = 2.3; CI (95%) = 1.19–4.44; *p* = 0.01).

**Fig. 1:**
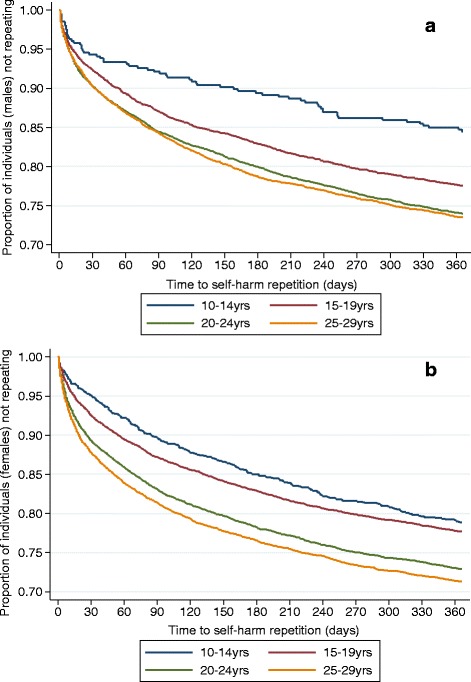
**a** Proportion of individuals (males) who did not repeat within 1-year follow-up period according to age. **b** Proportion of individuals (females) who did not repeat within 1-year follow-up period according to age

**Fig. 2:**
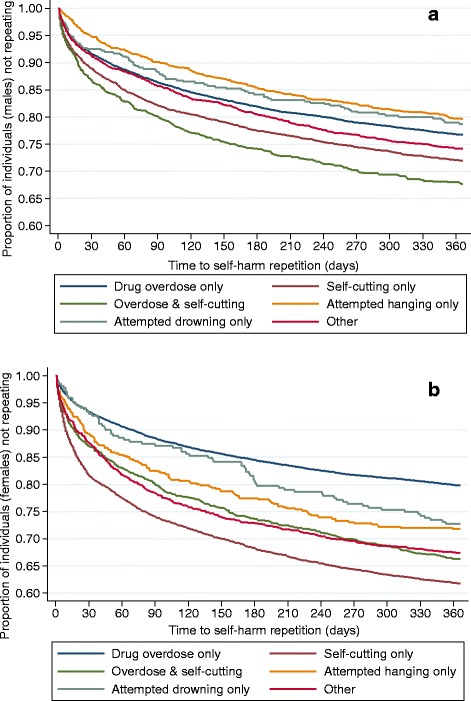
**a** Proportion of individuals (males) who did not repeat within 1-year follow-up period according to self-harm method. **b** Proportion of individuals (females) who did not repeat within 1-year follow-up period according to self-harm method

The results of Kaplan-Meier analyses are shown in Figs. 1a, b and 2a, b. Among all age groups risk of repetition was higher during the first months after the initial presentation (Fig. 1a and b). The proportions of those male patients who did not repeat within 3 months after a first episode were as follows: 92.0% in CH, 87.0% in AD, 84.0% in YEA, and 84.0% in OEA, respectively. Those male patients who did not repeat within 12 months after a first episode were as follows: 84.0% in CH, 77.5% in AD, 74.0% in YEA, and 73.5% in OEA. The proportions of those female patients who did not repeat within 3 months after a first episode were as follows: 90.0% in CH, 87.0% in AD, 83.0% in YEA, and 81.5% in OEA, respectively. Those female patients who did not repeat within 12 months after a first episode were as follows: 79.0% in CH, 78.0% in AD, 73.0% in YEA, and 71.5% in OEA, respectively.

The risk of repetition within 12 months after a first episode for those who presented due to self-cutting alone or combined with drug overdose was higher than that for individuals presenting due to other methods (Fig. 2a and b). According to the self-harm method used, the proportion of patients who did not repeat within 12 months after a first episode was as follows: in males, 68.0% for self-cutting combined with drug overdose; 72.0% for self-cutting (only); 77.0% for drug overdose (only); 79.0% for attempted drowning (only), and 79.5% for attempted hanging (only). In females, 62.0% for self-cutting (only); 66.0% for self-cutting combined with drug overdose; 72.0% for attempted hanging (only), 72.5% for attempted drowning (only), and 80.0% for drug overdose (only).

The risk of repetition increased with the number of previous self-harm episodes both in males and females. The proportions of male and female patients who did not repeat a self-harm act according to their self-harm history were similar within 1 year after a first presentation. Overall, the proportions of those patients who did not repeat within 12 months after a first episode was 86.0, 68.0, 54.5, 45.0, 35.0, and 37.0% for those with no, one, two, three, four and five previous episodes.

### Time between first presentations

Time between first and second presentations (T1-T2) was compared to time between second and third presentations (T2-T3). A comparison between low frequency repeaters (HFR) and high frequency repeaters (LFR), whose first presentation occurred during 2010–2013 showed some different patterns. Among LFR (*N* = 1088) T1-T2 (221 days) and T2-T3 (263 days) differed, that is an increase of 42 days was observed (CI (95%) = 8.81–76.86; *p* = 0.015). An opposite pattern was observed among HFR (*N* = 1485), a decrease of 47 days was observed (T1-T2 = 203 days, T2-T3 = 156 days) (CI (95%) = −75.59– −19.27; *p* = 0.001).

#### Age group

Comparisons by age group were conducted. Among adolescent HFR (*N* = 513), T1-T2 and T2-T3 differed (CI (95%) = −106.01– −10.99; *p* = 0.016). Among young emerging adult LFR (*N* = 392), T1-T2 and T2-T3 differed (CI (95%) = 1.98–117.84; *p* = 0.043). Among old emerging adult HFR (*N* = 390), T1-T2 and T2-T3 differed (CI (95%) = −111.18– −14.94; *p* = 0.01).

#### Gender, alcohol involvement, self-harm method

Among female LFR, T1-T2 (219 days) and T2-T3 (270 days) differed (CI (95%) = 3.64–99.41; *p* = 0.035). Among male HFR these periods differed (T1-T2 = 216; T2-T3 = 145 days; CI (95%) = −111.77– −30.77; *p* = 0.001).

When alcohol was involved in the act, among LFR T1-T2 and T2-T3 differed with an increase of 76 days (199; 275 days respectively; CI (95%) = 20.04–133.66; *p* = 0.008).

When drug overdose was involved in the act, among LFR T1-T2 and T2-T3 differed with and increase by 46 days (221; 267 days respectively; CI (95%) = 2.34–89.80; *p* = 0.039). Among HFR a decrease of 50 days was observed (CI (95%) = −89.44– −10.90; *p* = 0.012). Among LFR who used self-cutting (only; or with other methods) T1-T2 (189 days) differed from T2-T3 (251 days) (CI (95%) = 7.04–117.50; *p* = 0.027). Among HFR who used the same self-harm method T1-T2 (179 days) was only slightly different (marginal association) from T2-T3 (140 days) (CI (95%) = −81.33–1.50; *p* = 0.059).

Among females who used self-cutting (only; with other methods) comparisons by age group were conducted. Among adolescent LFR T1-T2 (128 days) differed from T2-T3 (266 days), CI (95%) = 38.40–238.80; *p* = 0.007). Among CH, YEA, and OEA these periods were similar.

## Discussion

This study investigated socio-demographic and self-harm characteristics of young individuals presenting to hospital emergency departments in Ireland due to self-harm. We have identified age similarities and differences in repetition of self-harm, focusing on frequency of repetitions and time between self-harm presentations. Our findings also highlight associations between repeated self-harm and socio-demographic and method characteristics and elucidate the relationship between recommended next care and repetition of self-harm across four age groups. We found that, among females, 15–19-year-old were at higher risk for repetition than 20–24-year-olds. Among females, those who used self-cutting were at higher risk for repetition than those who used intentional drug overdose. Overall, repeated self-harm risk increased significantly with the number of previous self-harm episodes. Time between second and third presentation increased compared to time between first and second presentation among low frequency repeaters, whereas the same time period decreased among high frequency repeaters.

We have investigated the relationship between socio-demographic and self-harm act characteristics and risk for repeated self-harm. Overall, age was not associated with repetition. However, among female patients, those aged 15–19 years old were at higher risk for repeated self-harm compared to those aged 20–24 years old. IJ Perry, et al. [17] reported that in females of all ages the peak rates of self-harm was in 15–19-year-old age group.

Overall, the risk for repetition for males and females was similar. However, among 20–24-year-old individuals, males more often presented to ED due to self-harm than females. A recent systematic review [28], which investigated hospital-based repeated self-harm, did not include gender among risk factors for repetition. Even though gender did not represent a risk factor for repetition in all age groups, our findings reveal that younger emerging adult males were at higher risk for repetition than females.

With regards to self-harm method used, female patients using self-cutting, only or combined with drug overdose, were at higher risk for subsequent self-harm compared to those using drug overdose only. This confirmed the findings of a study of Lilley et al. [29] which investigated self-harm presentations at ED in individuals of all age groups. The findings of Lilley showed that 47% of those who had used self-cutting presented again to EDs due to a self-harm act, compared to 31% of those who had used self-poisoning.

We found that risk of repetition increased with the number of previous self-harm episodes, this is in keeping with findings from previous studies investigating self-harm in young people [13] and individuals of all age groups [17].

Results from the present study suggest that repeated self-harm is associated with recommended next care only among some subgroups of patients. In particular, 20–24-year-old males admitted to general ward were at lower risk for repetition than those who left without being seen, and 25–29-year-old females admitted to general ward were at lower risk for repetition than those who were not admitted, were refused to be admitted and left before admission. Receiving after care following a self-harm episode is needed for every single patient presenting to ED. This is even more urgent among 20–24-year-old as they represent the subgroup with the highest rates among males [17]. It is relevant for every patient to receive an appropriate assessment as non-assessed patients are at greater risk of further self-harm and completed suicide than those who are assessed [30].

Observing subgroups of patients classified by total number of presentations by individual within 1 year following a first presentation revealed relevant findings. The proportion of females was overall higher than males, which confirmed the outcomes of studies in which Irish [31] and international samples [5] were analysed.

In addition to this, from some exploratory analyses, we found that the proportion of those using self-cutting compared to other methods was approximately 80% higher among those who had multiple episodes compared to those who had only one episode (17.9% of total number of presentations among those who presented once accounted for self-cutting, whereas 31.1% among those who presented seven or more times accounted for the same method). Conversely, the proportion of presentations by all other methods decreased among individuals with increasing number of presentations. This outcome confirmed the findings of K Hawton, et al. [13] who investigated self-harm among 10–18 year-old individuals and showed that self-cutting is a self-harm method associated with higher risk of repeated self-harm and suicide compared to self-poisoning.

To better identify people at higher risk for repetition, it was also relevant to examine the time between presentations at ED. This study found that time between second and third presentation was 42 days longer than time between first and second presentation among those patients who subsequently had a low number of repetitions, whereas this period was 47 days shorter among those who subsequently had a higher number of repetitions. The length of time between first presentations was shorter over time for those who subsequently presented to ED many times, and conversely longer for those who subsequently presented three times only. This pattern was confirmed in a number of subgroups of patients, that is both in adolescent and older emerging adult high frequency repeaters (from 3 to over 30 repetitions), and younger emerging adult low frequency repeaters (2 repetitions). The same pattern was found among female low frequency repeaters and male high frequency repeaters. Moreover, a similar pattern was also found among those who used drugs (overdose or other methods, both in LFR and HFR). Remarkably, among those who used self-cutting these time differences were even more marked compared to the general patient population. In fact, among low frequency repeaters using self-cutting the time difference between second and third presentation was 62 days longer than time between first and second presentation. Among adolescent low frequency repeater females who used self-cutting this pattern is highly marked, in fact the time difference between second and third presentation was 138 days longer than time between first and second presentation, whereas it was 42 days in the whole population.

A number of findings of this research confirm previous studies, others provide new information on at-risk individuals. Our results highlighted that self-harm repetition was more likely to occur among the given sub-groups of patients, that is younger emerging adult males, adolescent females, females using self-cutting, and those individuals with previous self-harm presentations.

Conducting the survival and the generalised estimated equation analysis was necessary to identify at-risk groups for self-harm repetition within 1 year following a first presentation. Survival analysis methods were used in previous similar studies [11, 13, 17, 24, 29], while estimated equation methods to assess first presentations time differences have innovatively employed in the current study.

### Strengths and limitations

The results of this study are robust as a large number of self-harm cases were analysed. Moreover, this study observed self-harm hospital presentations for a long time period. This represents a strength as not many studies to our knowledge investigating self-harm among young people have employed longitudinal data.

EM McMahon, et al. [31] showed that a large number of self-harm episodes are hidden, that is they do not come to the attention of health services. This study examined self-harm episodes involving presentation to ED only, not considering other cases. This may represent a limitation. However, the main objective of this research was to assess the specific patient population presenting to EDs due to self-harm, in order to inform ED services.

This study did not include possible suicide or other type of death following self-harm presentations which may have occurred during the follow-up period. However, as suicide is a rare event in young people in Ireland, particularly in adolescents [31], this has not affected the overall outcome of this study. The study could not take account of patients who presented to a hospital ED outside the Republic of Ireland. People who repeated and presented to a hospital ED other than an Irish hospital were not assessed. This may thus have slightly underestimated the rate of repetition.

The first presentation of the participants in the study period might not necessarily have been their first ever presentation at ED due to self-harm. However, in the survival analysis we excluded all of those patients who had presented between 1st January 2007 and 31st December 2009. It was thus unlikely they repeated after 3 or more years following a previous presentation. This is in accordance with current scientific literature which indicates that repeated self-harm in young people is most common in the first months following a presentation to ED [32].

## Conclusion

A number of subgroups of young patients presenting to hospital due to self-harm were at elevated risk of repeating a self-harm act within 1 year. Every person presenting at ED should receive a risk assessment to reduce the likelihood that such person represents over and over again with the same problem. Appropriate aftercare following a presentation due to self-harm should be put in place for everyone, irrespective of age. A health care professional should not underestimate the risk for a non-fatal or fatal suicidal act in young people. A common wrong belief might be that young people self-harm only temporarily and due to superficial reasons, such as a passing trend, or that improvements in young people are unlikely to occur due to ongoing physical and psychological development. These and other similar beliefs may hurt an individual in urgent need of help. Every health care provider dealing with self-harm and young people should go beyond these myths and be aware of the existence of non-suicidal self-injury disorder [33] and associated risks in order to best address the affected person’s needs. The findings of this study might help to inform a health care provider when conducting a risk assessment and planning next care. In particular, these findings might help to identify those individuals who are at higher risk for repeated self-harm. Additionally, the outcomes of this study highlight the importance of taking into account psychosocial characteristics, history of self-harm and the time between first presentations during risk assessment, as these represent indicators of risk of subsequent self-harm.

## References

[1] De Leo D, Heller TS (2004). Who are the kids who self-harm? An Australian self-report school survey. Med J Aust.

[2] Evans E, Hawton K, Rodham K (2005). In what ways are adolescents who engage in self-harm or experience thoughts of self-harm different in terms of help-seeking, communication and coping strategies?. J Adolesc.

[3] Laye-Gindhu A, Schonert-Reichl KA (2005). Nonsuicidal self-harm among community adolescents: understanding the “whats” and “whys” of self-harm. J Youth Adolesc.

[4] McMahon EM, Reulbach U, Corcoran P, Keeley HS, Perry IJ, Arensman E (2010). Factors associated with deliberate self-harm among Irish adolescents. Psychol Med.

[5] Evans E, Hawton K, Rodham K, Psychol C, Deeks J (2005). The prevalence of suicidal phenomena in adolescents: a systematic review of population-based studies. Suicide Life Threat Behav.

[6] Schmidtke A, Bille-Brahe U, DeLeo D, Kerkhof A, Bjerke T, Crepef P, Haring C, Hawton K, Lönnqvist J, Michel K (1996). Attempted suicide in Europe: rates, trend. S and sociodemographic characteristics of suicide attempters during the period 1989–1992. Results of the WHO/EURO Multicentre Study on Parasuicide. Acta Psychiatr Scand.

[7] Hawton K, Saunders KE, O’Connor RC (2012). Self-harm and suicide in adolescents. Lancet.

[8] Joiner TE, Conwell Y, Fitzpatrick KK, Witte TK, Schmidt NB, Berlim MT, Fleck M, Rudd MD (2005). Four studies on how past and current suicidality relate even when “everything but the kitchen sink” is covaried. J Abnorm Psychol.

[9] Carroll R, Metcalfe C, Gunnell D (2014). Hospital presenting self-harm and risk of fatal and non-fatal repetition: systematic review and meta-analysis. PLoS One.

[10] Bergen H, Hawton K, Waters K, Ness J, Cooper J, Steeg S, Kapur N (2012). How do methods of non-fatal self-harm relate to eventual suicide?. J Affect Disord.

[11] Zahl DL, Hawton K (2004). Repetition of deliberate self-harm and subsequent suicide risk: long-term follow-up study of 11 583 patients. Br J Psychiatry.

[12] Hawton K, James A (2005). Suicide and deliberate self harm in young people. BMJ.

[13] Hawton K, Bergen H, Kapur N, Cooper J, Steeg S, Ness J, Waters K (2012). Repetition of self-harm and suicide following self-harm in children and adolescents: findings from the Multicentre Study of Self-harm in England. J Child Psychol Psychiatry.

[14] Chitsabesan P, Harrington R, Harrington V, Tomenson B (2003). Predicting repeat self-harm in children. Eur Child Adolesc Psychiatry.

[15] Groholt B, Ekeberg Ø, Haldorsen T (2006). Adolescent suicide attempters: what predicts future suicidal acts?. Suicide Life Threat Behav.

[16] Hultén A, Jiang G-X, Wasserman D, Hawton K, Hjelmeland H, De Leo D, Ostamo A, Salander-Renberg E, Schmidtke A (2001). Repetition of attempted suicide among teenagers in Europe: frequency, timing and risk factors. Eur Child Adolesc Psychiatry.

[17] Perry IJ, Corcoran P, Fitzgerald AP, Keeley HS, Reulbach U, Arensman E (2012). The incidence and repetition of hospital-treated deliberate self harm: findings from the world’s first national registry. PLoS One.

[18] Health-Service-Executive: Securing the Future of Smaller Hospitals: A Framework for Development 2013. http://health.gov.ie/wp-content/uploads/2014/03/SecuringSmallerHospitals.pdf. Accesed 15 June 2015.

[19] Haw C, Bergen H, Casey D, Hawton K (2007). Repetition of deliberate self-harm: a study of the characteristics and subsequent deaths in patients presenting to a general hospital according to extent of repetition. Suicide Life Threat Behav.

[20] World-Health-Organization (2010). International statistical classification of diseases and related health problems 10th version.

[21] Cleves M. ssa13: Analysis of multiple failure-time data with Stata. Stata Technical Bulletin. 1999;49:30–39.

[22] Cleves M. How do I analyze multiple failure-time data using Stata2009. 1 August 2015.

[23] Prentice RL, Williams BJ, Peterson AV (1981). On the regression analysis of multivariate failure time data. Biometrika.

[24] Bergen H, Hawton K, Waters K, Cooper J, Kapur N (2010). Psychosocial assessment and repetition of self-harm: the significance of single and multiple repeat episode analyses. J Affect Disord.

[25] Hawton K, Bergen H, Casey D, Simkin S, Palmer B, Cooper J, Kapur N, Horrocks J, House A, Lilley R (2007). Self-harm in England: a tale of three cities. Soc Psychiatry Psychiatr Epidemiol.

[26] StataCorp (2011). Stata statistical software: release 12.

[27] IBM-Corp (2013). IBM SPSS statistics for Windows version 20.0.

[28] Larkin C, Di Blasi Z, Arensman E (2014). Risk factors for repetition of self-harm: a systematic review of prospective hospital-based studies. PLoS One.

[29] Lilley R, Owens D, Horrocks J, House A, Noble R, Bergen H, Hawton K, Casey D, Simkin S, Murphy E (2008). Hospital care and repetition following self-harm: multicentre comparison of self-poisoning and self-injury. Br J Psychiatry.

[30] Hickey L, Hawton K, Fagg J, Weitzel H (2001). Deliberate self-harm patients who leave the accident and emergency department without a psychiatric assessment: a neglected population at risk of suicide. J Psychosom Res.

[31] McMahon EM, Keeley H, Cannon M, Arensman E, Perry IJ, Clarke M, Chambers D, Corcoran P (2014). The iceberg of suicide and self-harm in Irish adolescents: a population-based study. Soc Psychiatry Psychiatr Epidemiol.

[32] Arensman E, Corcoran P, Fitzgerald AP (2011). Deliberate self-harm: extent of the problem and prediction of repetition. International Handbook of Suicide Prevention: Research, Policy and Practice.

[33] American-Psychiatric-Association: DSM 5: American Psychiatric Association; 2013.

